# Predicting the rapid progression of coronary artery lesions in patients with acute coronary syndrome based on machine learning

**DOI:** 10.3389/fcvm.2025.1535406

**Published:** 2025-09-05

**Authors:** Long Gui, Yuekang Hu, Hua Ouyang, Huanwei Zhuang, Yangfei Peng, Jun Yang, Heshan Cao, Songran Yang, Ping Hua

**Affiliations:** ^1^ Department of Cardiovascular Surgery, Sun Yat-sen Memorial Hospital, Sun Yat-sen University, Guangzhou, China; ^2^ Department of Cardiovascular Surgery, Haikou Affiliated Hospital of Central South University Xiangya School of Medicine, Haikou, China; ^3^ Department of Neurology, Sun Yat-sen Memorial Hospital, Sun Yat-sen University, Guangzhou, China; ^4^ Department of Biobank and Bioinformatics, Sun Yat-sen Memorial Hospital, Sun Yat-sen University, Guangzhou, China

**Keywords:** acute coronary syndrome, PCI, rapid progression, machine learning, random forest

## Abstract

**Purposes:**

Rapid coronary artery lesions (RCAL) are strongly linked to major adverse cardiovascular events in patients with acute coronary syndrome (ACS). This work developed a public online prediction platform for RCAL (OpRCAL) by comparing the performance of nine machine learning models.

**Methods:**

We retrospectively examined the clinical data of 324 patients with ACS who received percutaneous coronary intervention (PCI). Using both univariate and multivariate analyses, the potential independent risk factors for RCAL were studied. Following the screening of all variables using Lasso regression, multiple machine learning models were constructed. The optimal model was then chosen and validated using an external cohort. Furthermore, to elucidate the contribution of each feature to the model, the shapley additive explanation (SHAP) values of the variables were calculated. Finally, a prediction platform for RCAL in patients with ACS following PCI was established.

**Results:**

The number of coronary lesions, systolic blood pressure (SBP), N-terminal pro-brain natriuretic peptide (NT-proBNP), QRS interval, and platelet count were found as independent risk factors for RCAL. Among the nine machine learning models constructed after identifying twelve different variables using Lasso regression, the random forest (RF) model performed best in the training cohort and showed good generalization in the external test cohort, with area under curve of 0.774 (95%CI: 0.640–0.909). Finally, we constructed an online platform named OpRCAL for clinicians to predict RCAL in patients with ACS following PCI based on the RF model.

**Conclusions:**

The RF model exhibits high accuracy and generalizability in predicting RCAL, thereby providing a valuable instrument to assist clinical decision-making.

## 1 Introduction

Acute coronary syndrome (ACS) is a clinical syndrome caused by acute myocardial ischemia, with the pathological basis being the rupture of atherosclerotic plaques in the coronary arteries, leading to complete or incomplete occlusive thrombosis (1). The global registry of acute coronary events (GRACE) study showed that the mortality rate for ACS patients within one year of onset is approximately 15%, while the cumulative mortality rate over five years reaches as high as 20% (2, 3).

Nowadays, patients suffering from ACS have considerably better long-term prognoses owing to the widespread application of percutaneous coronary intervention (PCI). Nevertheless, coronary restenosis and progression of noncriminal vasculopathy can still develop in certain patients undergoing PCI, resulting in rapid coronary artery lesions (RCAL) (4, 5). The pathophysiology of restenosis following PCI, begins with endothelial injury produced by stent and balloon insertion. This injury triggers platelets activation, neointima proliferation, extracellular matrix formation, and vascular smooth muscle cells proliferation and migration (6). Microcalcification, extracellular matrix breakdown, and intra-plaque hemorrhage (IPH) are other mechanisms that may contribute to RCAL in non-target arteries. Previous studies have found that RCAL after PCI, whether occurring in target or non-target vessels, can lead to serious major adverse cardiovascular events (MACEs) (7). Therefore, a major problem for cardiologists is to reveal risk factors for RCAL as well as to identify the patients at high risk of developing RCAL.

Traditional risk factors for atherosclerosis, such as smoking, hypertension, diabetes, hyperlipidemia, and obese eating habits, are closely related to the occurrence of RCAL (8–10). Furthermore, both the severity of the lesion and the condition of the coronary artery are associated with RCAL development. For instance, clinical studies have shown that complex coronary artery morphology, intramural bleeding, and punctate calcification due to coronary spasm tend to advance more rapidly and severely than general lesions (11). In clinical practice, however, RCAL prediction and evaluation methods remain insufficient, as most studies on the subject have mainly focused on its related risk factors, with inconsistent results. Machine learning (ML) technology has found extensive usage in clinical practice due to its ability to fully exploit a variety of clinical variables for the purposes of disease screening, diagnosis, risk stratification, and prognosis evaluation (12–16).

In this study, we conducted a multicenter retrospective study that included clinical data from patients with ACS undergoing PCI, as well as follow-up visits. Our goal was to develop an easily accessible clinical tool to predict the incidence of RCAL following PCI using ML's algorithm.

## 2 Methods

### 2.1 Study population

This continuous retrospective cohort study included patients with ACS who received PCI treatment between June 2018 and June 2023 from two different centers. One center was Sun Yat-sen Memorial Hospital at Sun Yat-sen University, where the patients were enrolled in the training cohort, and another was the Haikou Affiliated Hospital of Central South University Xiangya School of Medicine, where the data were incorporated into the external test cohort. The study complied with all applicable regulations and standards, including those outlined in the requirements of the Declaration of Helsinki and the International Ethical Guidelines for Research Involving Human Health. It was approved by the Ethics Committee of Sun Yat-Sen Memorial Hospital (Ethics approval number: SYSEC-KY-KS-2023-253) and the Haikou Affiliated Hospital of Central South University Xiangya School of Medicine (Ethics approval number: SC20240504). Due to the retrospective nature of our methodology, the ethics committees waived the necessity for informed consent.

### 2.2 Inclusion-exclusion criteria

All patients with ACS in both the training and test cohorts required to meet the following criterion concurrently: (1) Study participants must meet the diagnostic criteria for ACS. (2) Patients had their first PCI and postoperative follow-up coronary angiography (CAG) examinations at the same medical institution. We excluded patients using the following criteria: (1) Age <18 years; (2) Missing or poor quality images; (3) Incomplete clinical data; (4) Intervals between first PCI and subsequent CAG examination <3 months; (5) Comorbidities with other structural cardiac disorders (e.g., congenital heart disease, cardiac large-vessel disease, cardiomyopathy, and valvular heart disease); and (6) A history of previous cardiac surgery (e.g., coronary artery bypass grafting, heart valve replacement, and transcatheter radiofrequency ablation).

### 2.3 Data collection

We collected 52 variables from each participant and divided them into four domains. The specifics are as follows: (1) General demographic characteristics, such as age, gender, body mass index, blood pressure, heart rate, and so forth; (2) Medications taken, including nitrates, calcium channel blockers, antiplatelet agents, lipid-regulating medications, etc.; (3) Laboratory blood tests, including red blood cell count, platelet count (PLT), creatinine, uric acid, lipids, fasting blood sugar, etc.; (4) Cardiovascular auxiliary examination, including echocardiography, CAG, and electrocardiogram.

### 2.4 Analysis of independent risk factors for RCAL

The diagnostic criteria for RCAL were derived from the references presented by Kaski and Zouridakis et al. (17, 18). Specifically, during the one-year follow-up period after PCI, one of the following requirements had to be satisfied: (1) A diameter reduction of 50% or more in previously successfully dilated lesions; (2) A diameter reduction of at least 10% in lesions with a pre-existing stenosis of 50% or more; (3) A diameter reduction of at least 30% in lesions with a pre-existing stenosis less than 50%; (4) New stenosis detected with a diameter reduction of 30% or more in segments of the vessel that were normal at the time of the first examination; and (5) Any lesion that progressed to complete occlusion on the second examination. The quantitative evaluation of the first and follow-up CAG images was conducted by a cardiologist with extensive experience in the evaluation of coronary lesion progression. According to the RCAL diagnosis, participants in the training cohort were divided into progressive and non-progressive groups. Subsequent univariate and multivariate regression analyses were conducted to identify the independent risk factors for RCAL.

### 2.5 Variable selection

Lasso regression was used to automatically identify the most significant features by reducing the coefficients of irrelevant features to zero. This prevented overfitting, which improved the model's generalization, and made the model more concise and comprehensible. After acquiring the variables for model fitting using Lasso regression, we employed the variance inflation factor (VIF) to check for multicollinearity between variables.

### 2.6 Construction and evaluation of ML models

In this study, the training cohort was split into an internal training cohort and an internal validation cohort, in a ratio of 8:2. The distribution of progressive and non-progressive instances in the divided dataset were balanced using stratified sampling. Continuous variables were normalized, while categorical variables were encoded using a specific one-hot encoding approach. We developed nine ML models, including decision trees (DT), elastic net (ENET), k-nearest neighbor (KNN), light gradient boosting machine (LightGBM), logistic regression (LR), multilayer perceptron (MLP), random forest (RF), support vector machine (SVM), and extreme gradient boosting (XGBoost). To identify the optimal hyperparameters for each model, we used internal 5-fold cross-validation and grid search. The models' performance was carefully evaluated, considering the average area under the ROC curve, accuracy, sensitivity, specificity, and other relevant metrics. The model with the best average performance was selected as the final prediction method and tested with an external cohort. The mean reduction in Gini coefficient and shapley additive explanation (SHAP) values were used to evaluate the significance of the variables in the model we developed. The mean Gini coefficient reduction is a metric that determines the extent to which each variable contributes to the homogeneity of the random forest's nodes and leaves. A higher value indicates that the variable is of higher precedence for inclusion in the model. The SHAP values were then calculated to further elucidate the contribution of each feature to the model. This information was then used to establish a practicable and straightforward prediction platform for RCAL after PCI.

### 2.7 Statistical methods

Data analysis and visualization were conducted using SPSS (version 23.0), R Studio (version 4.2.3), and Shiny (version 1.8.1.1). Categorical variables were assessed using the chi-square test or Fisher's exact test, and the results were reported as absolute numbers and percentages. Continuous variables with a normal distribution were presented as mean ± standard deviation and examined using *t*-test. Data that didn't conform to a normal distribution were described using quartiles and evaluated using nonparametric tests. Multiple groups of continuous variables with normal distributions were analyzed using one-way ANOVA, whereas those with skewed distributions were evaluated using the Kruskal–Wallis H test. Statistical significance was defined as a two-sided *p*-value of less than 0.05.

## 3 Results

### 3.1 Distribution of clinical data of the study population

The training cohort included 324 patients with a median age of 64 (57, 70) years, with 227 (70.06%) being male. During the follow-up period, 84 (25.93%) patients developed RCAL (Table 1). After allocation in an 8:2 ratio, the internal training cohort included 259 individuals (25.90% of RCAL events) and the internal validation cohort had 65 individuals (26.20% of RCAL events). The external test cohort included 63 patients with a median age of 65 (58, 70) years, of whom 46 (73.00%) were males, and RCAL occurred in 19 (30.20%) during follow-up (Table 2).

**Table 1:** Demographic and clinical characteristics of patients in the training cohort.

Characteristic	Total (*n* = 324)	Non-progress group (*n* = 240)	Progress group (*n* = 84)	*P*-value
General Demographic Characteristics				
Gender (male)	227 (70.06%)	165 (68.75%)	62 (73.81%)	0.384
Age (year)	64.00 (57.00, 70.00)	64.00 (57.00, 70.00)	65.00 (57.75, 71.00)	0.427
Weight (kg)	65.67 ± 10.90	65.57 ± 10.71	65.97 ± 11.49	0.770
Height (cm)	164.00 (156.00, 170.00)	163.50 (156.75, 169.00)	164.50 (156.00, 170.00)	0.576
BMI (kg/m^2^)	24.30 (22.49, 26.91)	24.37 (22.48, 26.91)	24.13 (22.57, 26.93)	0.880
SBP (mmHg)	132.50 (120.00, 145.25)	131.00 (118.75, 141.00)	140.50 (125.75, 151.25)	<0.001
DBP (mmHg)	80.00 (73.00, 86.00)	80.00 (72.75, 84.00)	80.50 (74.50, 89.00)	0.066
HR (bpm)	71.00 (62.00, 79.00)	71.00 (62.00, 79.00)	71.00 (61.75, 79.25)	0.773
Hypertension (Yes)	206 (63.58%)	140 (58.33%)	66 (78.57%)	<0.001
Diabetes (Yes)	80 (24.69%)	53 (22.08%)	27 (32.14%)	0.066
Hyperlipemia (Yes)	63 (19.44%)	50 (20.83%)	13 (15.48%)	0.286
Smoking (Yes)	132 (40.74%)	96 (40.00%)	36 (42.86%)	0.646
Drinking (Yes)	48 (14.81%)	37 (15.42%)	11 (13.10%)	0.606
History of Drug Use				
Aspirin (Yes)	161 (49.69%)	121 (50.42%)	40 (47.62%)	0.659
Clopidogrel or Ticagrelor (Yes)	220 (67.90%)	166 (69.17%)	54 (64.29%)	0.410
Lipid-lowering drug (Yes)	247 (76.23%)	187 (77.92%)	60 (71.43%)	0.229
Nitrates (Yes)	100 (30.86%)	73 (30.42%)	27 (32.14%)	0.768
CCB (Yes)	116 (35.80%)	85 (35.42%)	31 (36.90%)	0.807
ACEI (Yes)	47 (14.51%)	28 (11.67%)	19 (22.62%)	0.014
ARB (Yes)	78 (24.07%)	57 (23.75%)	21 (25.00%)	0.818
*β*-blocker (Yes)	144 (44.44%)	104 (43.33%)	40 (47.62%)	0.496
Diuretic (Yes)	33 (10.19%)	20 (8.33%)	13 (15.48%)	0.062
Laboratory Blood Tests				
WBC (10^9^/L)	7.12 (6.10, 8.48)	7.12 (6.01, 8.32)	7.31 (6.47, 9.13)	0.125
RBC (10^12^/L)	4.56 (4.27, 4.84)	4.56 (4.27, 4.83)	4.58 (4.33, 4.94)	0.233
HGB (g/L)	137.00 (128.00, 145.00)	137.00 (128.00, 145.00)	137.00 (127.00, 146.00)	0.888
PLT (10^9^/L)	237.00 (201.00, 270.00)	232.00 (188.00, 256.00)	273.00 (228.00, 304.25)	<0.001
HCT (%)	41.00 (38.00, 43.00)	41.00 (38.00, 43.00)	41.00 (39.00, 43.00)	0.997
UA (μmol/L)	399.94 ± 109.54	398.27 ± 111.10	404.70 ± 105.44	0.644
Cr (μmol/L)	88.00 (77.00, 102.00)	88.00 (76.75, 101.00)	89.50 (78.50, 109.75)	0.295
CHOL (mmol/L)	4.87 ± 1.23	4.81 ± 1.23	5.03 ± 1.24	0.159
TG (mmol/L)	1.49 (1.07, 2.08)	1.52 (1.04, 2.08)	1.41 (1.10, 2.05)	0.923
HDL-C (mmol/L)	1.07 (0.91, 1.24)	1.08 (0.92, 1.24)	1.04 (0.88, 1.23)	0.502
LDL-C (mmol/L)	3.06 (2.44, 3.75)	3.01 (2.37, 3.67)	3.21 (2.59, 4.00)	0.081
ApoA1 (g/L)	1.14 (1.01, 1.26)	1.14 (1.01, 1.27)	1.12 (1.00, 1.23)	0.202
ApoB (g/L)	0.94 (0.75, 1.09)	0.94 (0.74, 1.08)	0.94 (0.77, 1.10)	0.252
hs-CRP (mg/L)	2.08 (1.11, 5.79)	2.08 (1.02, 5.15)	2.64 (1.25, 7.42)	0.076
FBS (mmol/L)	5.00 (4.60, 5.80)	5.10 (4.60, 5.80)	5.00 (4.50, 6.10)	0.813
NT-proBNP (pg/ml)	89.40 (42.08, 269.23)	71.90 (37.05, 178.67)	184.70 (80.40, 654.08)	<0.001
FFA (mmol/L)	416.00 (334.25, 546.00)	416.00 (334.25, 546.50)	416.00 (338.75, 528.25)	0.977
CPK (U/L)	91.00 (70.00, 130.50)	91.00 (70.00, 125.25)	96.50 (68.50, 141.00)	0.721
LDH (U/L)	178.00 (159.00, 208.00)	178.00 (159.00, 207.00)	179.00 (160.00, 209.25)	0.611
CKMB (U/L)	12.00 (9.00, 16.00)	12.00 (10.00, 15.00)	11.50 (7.00, 16.00)	0.059
Cardiovascular Auxiliary Examination				
LVEF (%)	67.00 (63.00, 71.25)	68.00 (63.75, 72.00)	65.00 (61.00, 70.00)	0.002
LAD (mm)	34.00 (31.00, 37.00)	33.00 (31.00, 36.00)	36.00 (32.00, 39.00)	<0.001
LVDd (mm)	48.00 (45.00, 51.00)	48.00 (45.00, 50.00)	49.00 (45.00, 52.25)	0.112
LVPWd (mm)	9.00 (9.00, 10.00)	9.00 (9.00, 10.00)	10.00 (9.00, 11.00)	0.003
IVSd (mm)	10.00 (9.00, 11.00)	9.00 (9.00, 10.00)	10.00 (9.00, 11.00)	0.004
RVDd (mm)	20.00 (19.00, 22.00)	20.00 (19.00, 21.00)	21.00 (19.75, 23.00)	0.017
Number of coronary lesions	2.00 (1.00, 3.00)	2.00 (1.00, 3.00)	3.00 (2.00, 3.00)	<0.001
PR interval (ms)	160.00 (150.00, 176.00)	160.00 (150.00, 178.00)	160.00 (149.00, 174.00)	0.908
QRS interval (ms)	88.00 (80.00, 95.25)	86.00 (80.00, 94.00)	90.00 (84.75, 98.00)	0.001
QT interval (ms)	380.00 (360.00, 400.00)	380.00 (360.00, 400.00)	388.00 (354.25, 408.00)	0.220

BMI, body mass index; SBP, systolic blood pressure; DBP, diastolic blood pressure; HR, heart rate; CCB, calcium channel blockers; ACEI, angiotensin converting enzyme inhibitors; ARB, angiotensin receptor blockers; WBC, white blood cell; RBC, red blood cell; HGB, hemoglobin; PLT, platelet; HCT, hematocrit; UA, uric acid; Cr, greatinine; CHOL, cholesterol; TG, triglyceride; HDL-C, high density lipoprotein cholesterol; LDL-C, low density lipoprotein cholesterol; ApoA1, apolipoprotein A1; ApoB, apolipoprotein B; hs-CRP, high sensitivity C reactive protein; FBS, fasting blood sugar; NT-proBNP, N-terminal pro-brain natriuretic peptide; FFA, free fatty acids; CPK, creatinine phosphkinaes; LDH, lactate dehydrogenase; CKMB, creatine kinase isoenzymes; LVEF, left ventricular ejection fraction; LAD, left atrium diameter; LVDd, left ventricular end-diastolic diameter; LVPWd, left ventricular posterior wall diastolic thickness; IVSd, interventricular septal end-diastolic thinkness; RVDd, right ventricular end-diastolic diameter.

**Table 2:** Demographic and clinical characteristics of internal training cohort, internal validation cohort, and external test cohort.

Characteristic	Internal training cohort (*n* = 259)	Internal validation cohort (*n* = 65)	External test cohort (*n* = 63)	*P*-value
General Demographic Characteristics				
Gender (male)	186 (71.80%)	41 (63.10%)	46 (73.00%)	0.346
Age (year)	65.00 (58.00, 71.00)	62.00 (57.00, 68.00)	65.00 (58.00, 70.00)	0.264
Weight (kg)	65.51 ± 10.64	66.32 ± 11.97	66.88 ± 11.76	0.635
Height (cm)	164.00 (157.00, 170.00)	162.00 (155.50, 168.50)	165.00 (160.00, 170.00)	0.056
BMI (kg/m^2^)	24.20 (22.49, 26.66)	25.34 (22.85, 27.91)	24.49 (21.89, 26.43)	0.225
SBP (mmHg)	132.00 (119.00, 146.00)	133.00 (124.00, 144.00)	133.00 (121.00, 145.00)	0.958
DBP (mmHg)	79.24 ± 10.71	80.42 ± 10.06	77.48 ± 11.33	0.293
HR (bpm)	71.00 (63.00, 79.00)	71.00 (61.50, 76.50)	71.00 (63.00, 82.00)	0.670
Hypertension (Yes)	165 (63.70%)	41 (63.10%)	33 (52.40%)	0.254
Diabetes (Yes)	67 (25.90%)	13 (20.00%)	23 (36.50%)	0.095
Hyperlipemia (Yes)	51 (19.70%)	12 (18.50%)	22 (34.90%)	0.023
Smoking (Yes)	109 (42.10%)	23 (35.40%)	21 (33.30%)	0.333
Drinking (Yes)	37 (14.30%)	11 (16.90%)	17 (27.00%)	0.056
Group (Progress group)	67 (25.90%)	17 (26.20%)	19 (30.20%)	0.806
History of Drug Use				
Aspirin (Yes)	126 (48.60%)	35 (53.80%)	59 (93.70%)	<0.001
Clopidogrel or Ticagrelor (Yes)	175 (67.60%)	45 (69.20%)	45 (71.40%)	0.845
Lipid-lowering drug (Yes)	193 (74.50%)	54 (83.10%)	60 (95.20%)	0.001
Nitrates (Yes)	91 (35.10%)	9 (13.80%)	43 (66.70%)	<0.001
CCB (Yes)	96 (37.10%)	20 (30.80%)	16 (25.40%)	0.180
ACEI (Yes)	36 (13.90%)	11 (16.90%)	17 (27.00%)	0.041
ARB (Yes)	65 (25.10%)	13 (20.00%)	20 (31.70%)	0.316
β-blocker (Yes)	111 (42.90%)	33 (50.80%)	27 (42.90%)	0.516
Diuretic (Yes)	28 (10.80%)	5 (7.70%)	17 (27.00%)	0.001
Laboratory Blood Tests				
WBC (10^9/L)	7.12 (5.99, 8.56)	7.12 (6.14, 8.40)	7.46 (5.36, 10.72)	0.850
RBC (10^12/L)	4.56 (4.27, 4.84)	4.56 (4.29, 4.92)	4.53 (4.13, 4.73)	0.558
HGB (g/L)	137.00 (127.00, 145.00)	136.00 (128.50, 147.00)	131.00 (119.00, 139.00)	0.011
PLT (10^9/L)	239.00 (201.00, 271.00)	233.00 (199.50, 269.00)	223.00 (200.00, 269.00)	0.456
HCT (%)	41.00 (38.00, 43.00)	41.00 (38.00, 44.00)	39.30 (35.60, 43.00)	0.170
UA (μmol/L)	88.00 (78.00, 101.00)	87.00 (72.50, 107.50)	98.00 (77.00, 117.00)	0.327
Cr (μmol/L)	386.00 (326.00, 462.00)	402.00 (299.50, 510.00)	309.00 (261.00, 383.00)	<0.001
CHOL (mmol/L)	4.70 (3.92, 5.75)	4.87 (4.06, 5.42)	4.65 (3.75, 5.61)	0.723
TG (mmol/L)	1.49 (1.04, 2.02)	1.49 (1.09, 2.29)	1.39 (0.85, 2.20)	0.794
HDL-C (mmol/L)	1.06 (0.91, 1.24)	1.08 (0.88, 1.25)	1.13 (0.94, 1.51)	0.043
LDL-C (mmol/L)	3.10 (2.44, 3.80)	3.04 (2.48, 3.53)	2.88 (2.09, 3.39)	0.153
ApoA1 (g/L)	1.14 (1.00, 1.27)	1.14 (1.02, 1.22)	1.20 (1.00, 1.40)	0.081
ApoB (g/L)	0.94 (0.74, 1.10)	0.93 (0.76, 1.04)	1.00 (0.69, 1.25)	0.350
hs-CRP (mg/L)	2.08 (1.01, 6.23)	2.10 (1.32, 5.51)	2.59 (1.12, 5.86)	0.755
FBS (mmol/L)	5.10 (4.60, 5.80)	4.80 (4.45, 5.85)	5.44 (4.57, 6.06)	0.211
NT-proBNP (pg/ml)	86.80 (42.00, 280.50)	102.00 (40.80, 241.65)	109.40 (55.10, 417.50)	0.272
FFA (mmol/L)	416.00 (338.00, 546.00)	416.00 (313.50, 559.00)	520.00 (350.00, 620.00)	0.027
CPK (U/L)	91.00 (72.00, 126.00)	91.00 (65.00, 155.00)	114.00 (83.00, 558.00)	0.009
LDH (U/L)	178.00 (159.00, 208.00)	176.00 (157.50, 204.50)	204.00 (154.00, 315.00)	0.038
CKMB (U/L)	12.00 (9.00, 15.00)	12.00 (9.00, 16.00)	11.00 (6.60, 35.30)	0.952
Cardiovascular Auxiliary Examination				
LVEF (%)	67.00 (63.00, 71.00)	66.00 (62.00, 72.00)	65.00 (60.00, 69.00)	0.058
LAD (mm)	34.00 (31.00, 37.00)	34.00 (32.00, 37.00)	33.00 (31.00, 35.00)	0.116
LVDd (mm)	48.00 (45.00, 51.00)	48.00 (46.00, 51.00)	43.00 (40.00, 49.00)	<0.001
LVPWd (mm)	9.00 (9.00, 10.00)	9.00 (9.00, 10.00)	10.00 (9.00, 11.00)	0.424
IVSd (mm)	10.00 (9.00, 11.00)	10.00 (8.50, 11.00)	11.00 (9.00, 12.00)	0.006
RVDd (mm)	20.00 (19.00, 22.00)	20.00 (19.00, 22.00)	25.00 (21.00, 29.00)	<0.001
Number of coronary lesions	2.00 (2.00, 3.00)	2.00 (1.00, 3.00)	3.00 (1.00, 3.00)	0.125
PR interval (ms)	160.00 (150.00, 176.00)	160.00 (150.00, 176.00)	156.00 (132.00, 172.00)	0.008
QRS interval (ms)	88.00 (80.00, 96.00)	88.00 (80.00, 94.00)	88.00 (82.00, 100.00)	0.777
QT interval (ms)	380.00 (359.00, 400.00)	382.00 (360.00, 402.00)	386.00 (352.00, 404.00)	0.735

BMI, body mass index; SBP, systolic blood pressure; DBP, diastolic blood pressure; HR, heart rate; CCB, calcium channel blockers; ACEI, angiotensin converting enzyme inhibitors; ARB, angiotensin receptor blockers; WBC, white blood cell; RBC, red blood cell; HGB, hemoglobin; PLT, platelet; HCT, hematocrit; UA, uric acid; Cr, greatinine; CHOL, cholesterol; TG, triglyceride; HDL-C, high density lipoprotein cholesterol; LDL-C, low density lipoprotein cholesterol; ApoA1, apolipoprotein A1; ApoB, apolipoprotein B; hs-CRP, high sensitivity C reactive protein; FBS, fasting blood sugar; NT-proBNP, N-terminal pro-brain natriuretic peptide; FFA, free fatty acids; CPK, creatinine phosphkinaes; LDH, lactate dehydrogenase; CKMB, creatine kinase isoenzymes; LVEF, left ventricular ejection fraction; LAD, left atrium diameter; LVDd, left ventricular end-diastolic diameter; LVPWd, left ventricular posterior wall diastolic thickness; IVSd, interventricular septal end-diastolic thinkness; RVDd, right ventricular end-diastolic diameter.

### 3.2 Clinically independent risk factors for RCAL

A total of 14 clinical variables were identified as risk factors for coronary progression in ACS in a univariate analysis, including a history of hypertension, use of angiotensin-converting enzyme inhibitor medication, the number of coronary lesions, systolic blood pressure (SBP), N-terminal pro-brain natriuretic peptide (NT-proBNP), and PLT count, and so on. The results of multivariate analysis revealed that the number of coronary artery lesions (HR = 1.55, 95% CI: 1.09–2.20, *p* = 0.014), SBP (HR = 1.02, 95% CI: 1.01–1.04, *p* = 0.006), NT-proBNP (HR = 1.01, 95% CI: 1.01–1.01, *p* = 0.001), QRS duration (HR = 1.02, 95% CI: 1.01–1.04, *p* = 0.034), and PLT count (HR = 1.02, 95% CI: 1.02–1.03, *p* < 0.001) were independent risk factors for RCAL in ACS (Supplementary Table S1).

### 3.3 Variable selection

**Figure 1:**
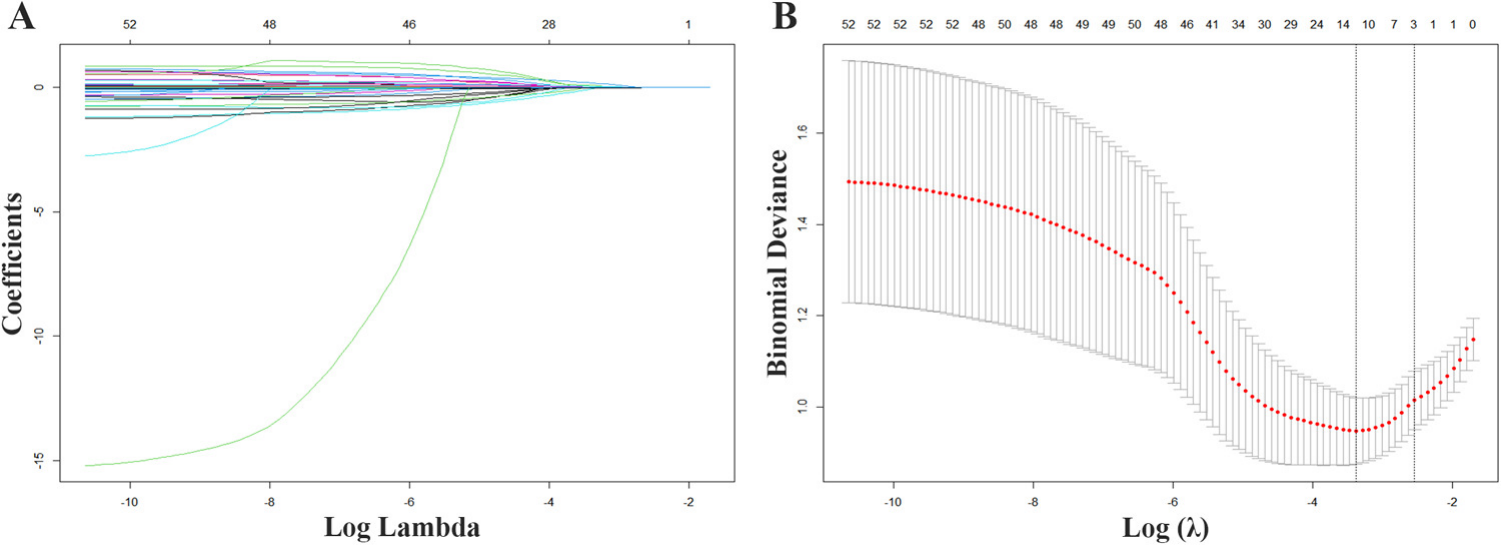
Variable screening with Lasso regression. **(A)** Lasso coefficient curves for 52 demographic and clinical features plotted from the log (*λ*) series. **(B)** The optimal parameter (lamda) selection for Lasso used 10-fold cross validation.

Figure 1 shows that 12 variables were discovered using Lasso regression analysis and ten-fold cross-validation, with the lowest mean error and the strongest coefficient matching to log (*λ*). According to the results of the multiple collinearity tests using VIF, all 12 variables were substantially independent of one another, with each having a VIF value of less than 3 and a tolerance greater than 0.2 (Supplementary Table S2). As a result, 12 variables were selected as features for the subsequent ML model construction: age, a history of hypertension, a history of hyperlipidemia, SBP, number of coronary lesions, NT-proBNP, left ventricular ejection fraction (LVEF), left atrium diameter (LAD), interventricular septal end-diastolic thinkness (IVSd), right ventricular end-diastolic diameter (RVDd), QRS interval, and PLT count.

### 3.4 Performance of multiple ML models

**Figure 2:**
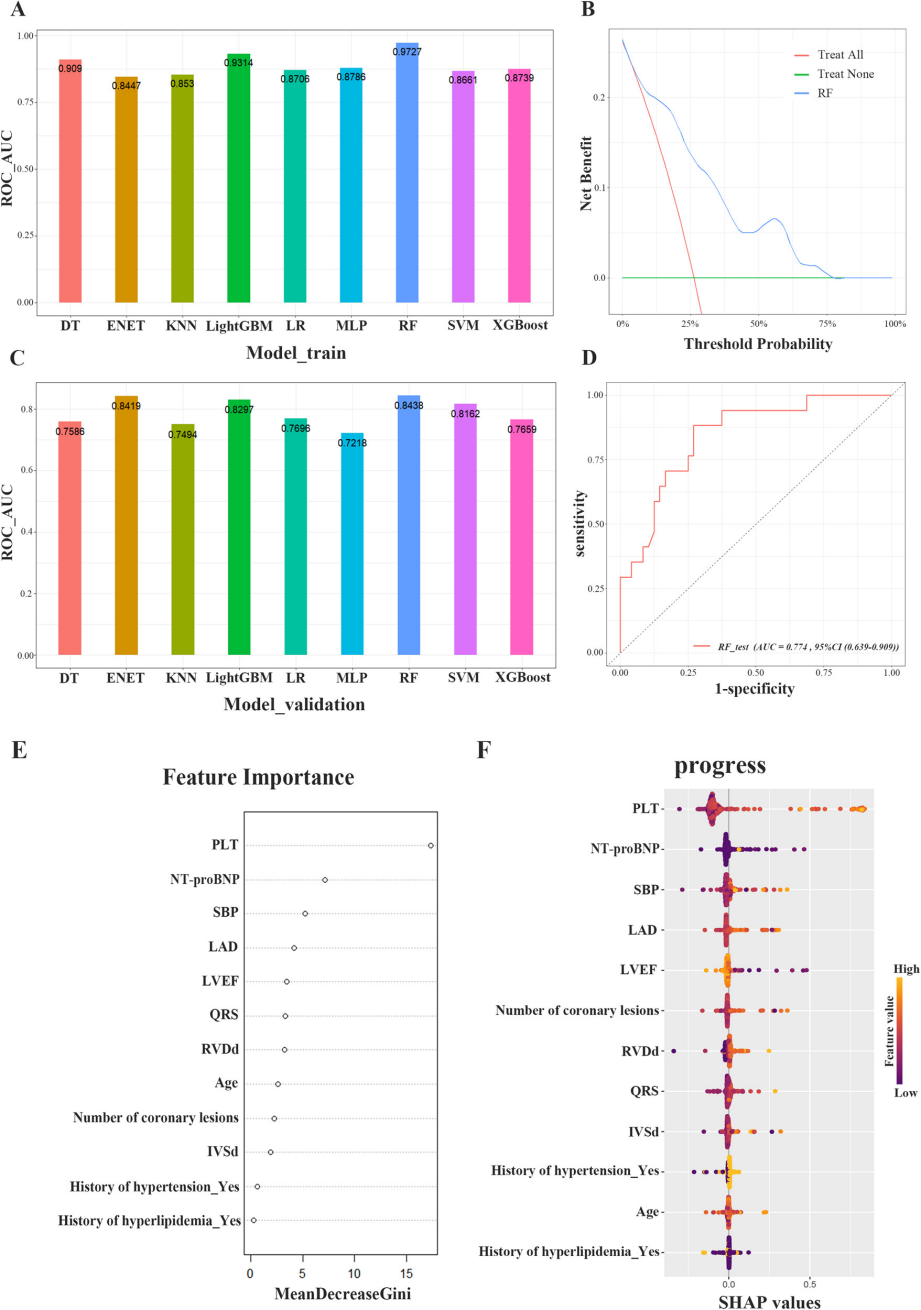
Evaluation of model performance. **(A)** Performance of nine machine learning models in the area under the ROC curve (AUC) of the internal training cohort. **(B)** Decision curve analysis (DCA) of RF model in the internal training cohort. **(C)** Performance of nine machine learning models in the area under the ROC curve (AUC) of the internal validation cohort. **(D)** ROC curve analysis of the RF algorithm for predicting rapid progression of coronary lesions in the external testing cohort. **(E)** The importance of the features included in the construction of the RF model. **(F)** SHAP analysis of the RF model for predicting rapid progression of coronary lesions. ROC, receiver operating characteristic curve; AUC, area under curve; DCA, decision curve analysis; RF, random forest; SHAP, shapley additive explanation.

We developed 9 ML models to predict RCAL. The area under curve (AUC) of the 9 models in the internal training cohort ranked as follows: RF (0.973), LightGBM (0.931), DT (0.909), MLP (0.897), XGBoost (0.874), LR (0.871), SVM (0.866), KNN (0.853) and ENET (0.845) (Figure 2A). The internal training cohort's top 5 accuracy rankings were: RF (0.931), LightGBM (0.880), DT (0.822), XGBoost (0.819) and MLP (0.819). In the internal validation cohort, the AUC of the 9 models were RF (0.844), ENET (0.842), LightGBM (0.830), SVM (0.816), LR (0.770), XGBoost (0.766), DT (0.759), KNN (0.749) and MLP (0.722), respectively (Figure 2C). The internal validation cohort's top 5 accuracy rankings were as follows: RF (0.800), LightGBM (0.800), SVM (0.738), XGBoost (0.738) and KNN (0.738). Additional performance metrics are presented in Table 3 and Supplementary Figure S. RF was chosen as the final prediction model for RCAL due to its superior performance in both the internal training and validation cohorts. The results of decision curve analysis (DCA) showed that the RF model had a maximum net benefit of 0.24 for threshold probability ranging from 0.03 to 0.78, highlighting its high performance (Figure 2B). Figure 2D showed that the RF model's performance was stable in the external test cohort, suggesting that it is highly generalizable (AUC = 0.774, 95% CI: 0.639–0.909).

**Table 3:** Performance comparison of each model in the internal training cohort and the internal validation cohort.

Cohort	Model	Accuracy (%)	F1 score (%)	NPV (%)	PPV (%)	Precision (%)	Recall (%)	AUC (%)	Sensitivity (%)	Specificity (%)
Internal training cohort	DT	82.24	72.29	95.63	60.61	60.61	89.55	90.90	89.55	79.69
	ENET	77.99	65.03	91.41	55.21	55.21	79.10	84.47	79.10	77.60
	KNN	74.13	63.78	94.33	50.00	50.00	88.06	85.30	88.06	69.27
	LightGBM	88.03	91.60	71.95	95.48	95.48	88.02	93.14	88.02	88.06
	LR	73.36	78.77	49.21	96.24	96.24	66.67	87.06	66.67	92.54
	MLP	81.85	86.98	61.11	92.90	92.90	81.77	87.86	81.77	82.09
	RF	93.05	95.16	81.01	98.33	98.33	92.19	97.27	92.19	95.52
	SVM	80.31	85.95	59.09	91.23	91.23	81.25	86.61	81.25	77.61
	XGBoost	81.85	68.46	90.96	62.20	62.20	76.12	87.39	76.12	83.85
Internal validation cohort	DT	69.23	56.52	88.89	44.83	44.83	76.47	75.86	76.47	66.67
	ENET	72.31	52.63	84.09	47.62	47.62	58.82	84.19	58.82	77.08
	KNN	73.85	56.41	86.05	50.00	50.00	64.71	74.94	64.71	77.08
	LightGBM	80.00	66.67	90.70	59.09	59.09	76.47	82.97	76.47	81.25
	LR	69.23	77.27	44.00	85.00	85.00	70.83	76.96	70.83	64.71
	MLP	72.31	55.00	85.71	47.83	47.83	64.71	72.18	64.71	75.00
	RF	80.00	86.02	60.00	88.89	88.89	83.33	84.38	83.33	70.59
	SVM	73.85	81.72	50.00	84.44	84.44	79.17	81.62	79.17	58.82
	XGBoost	73.85	82.11	50.00	82.98	82.98	81.25	76.59	81.25	52.94

NPV, negative predictive value; PPV, positive predictive value; AUC, area under the curve.

### 3.5 Variable importance and variable interpretation

Figure 2E showed that among the features used to build the model, PLT count, NT-proBNP, SBP, LAD, and LVEF ranked highest in terms of mean Gini coefficient reduction. The impact of each variable on the model's prediction was also demonstrated using SHAP values, as shown in Figure 2F.

### 3.6 Construction of online public platform

We achieved robust prediction outcomes using the RF model, by factoring in the importance of the variables and their combinations. Based on these findings, we developed a public online platform to facilitate individualized prediction of RCAL (OpRCAL) in patients with ACS after PCI. The platform can be accessed at: https://cardiovascular-surgery.shinyapps.io/progress_prediction/.

## 4 Discussion

In this study, we discovered five independent risk factors for RCAL: number of lesions, SBP, NT-proBNP, QRS duration, and PLT count, all of which were included in the 12 feature variables chosen using Lasso regression. We selected the RF model to predict RCAL based on the given feature variables since it outperformed the other 8 ML algorithms in both the internal training and validation cohorts and showed strong generalization in the external cohort. In light of these findings, we developed OpRCAL, a public online platform that enables individualized prediction of RCAL in patients with ACS following PCI.

ML has distinct benefits and is hence a preferred approach to building clinical prediction models. Our study included nine ML algorithms, with AUC and accuracy serving as the main measures of model performance. To reduce variability in model performance assessment, the final model was selected using the mean AUC value from the internal 5-fold cross-validation. The RF model achieved superior accuracy and AUC in both the internal training and validation cohorts. Impressively, it also surpassed competitors in terms of F1 score, positive predictive value, recall, and sensitivity. RF is an ensemble learning algorithm based on decision trees, that is widely favored in coronary artery disease research due to its excellent accuracy and robustness. Wang et al. (19) highlighted RF's capability in predicting premature coronary artery disease, offering valuable insights for early screening and clinical decision-making in disease prevention. Additionally, Hadanny et al. (20) shown that an RF-based survival prediction model outperformed deep learning and traditional statistical methods for predicting one-year mortality in patients with ACS. Our study reinforces these findings, demonstrating RF's effectiveness. Furthermore, we computed SHAP values for each variable and generated SHAP diagrams to make the RF model, which is notoriously a “black box”, easier to understand. Our study revealed that that PLT count, NT-proBNP, and SBP play significant roles in the RF model.

We found that PLT count is an independent risk factor for RCAL. The progress group had significantly higher baseline PLT count compared to the non-progress group [232.00 (188.00, 256.00) vs. 273.00 (228.00, 304.25), *p* < 0.05]. However, there were no significant differences in the use of cyclo-oxygenase inhibitor aspirin [121 (50.42%) vs. 40 (47.62%), *p* = 0.659], or diphosphate receptor antagonist clopidogrel or ticagrelor [166 (69.17%) vs. 54 (64.29%), *p* = 0.410], when comparing the two groups' antiplatelet medication usage. It is currently believed that PLT plays an important role in the evolution of atherosclerotic plaques. Following endothelial injury, PLT adheres to the exposed subendothelium, releases vasoactive substances, and promotes smooth muscle cell migration and proliferation (21). In addition, PLT can enhance foam cell formation even in patients without hyperlipidemia (22), and it may serve as an important lipid supply for lipid pattern formation (23). What's more, it was demonstrated that PLT can accelerate atherosclerosis formation by regulating the expression levels of cytokines like SOCS3 and IL-1β, inducing monocyte macrophages to produce inflammatory features, and promoting monocyte recruitment at plaques (24).

Our findings indicate that high blood NT-proBNP expression at baseline is positively associated with RCAL. NT-proBNP is an established prognostic biomarker for cardiovascular events in patients with ACS and stable coronary artery disease (25, 26). Several pathological conditions have been linked to higher blood NT-proBNP levels in ACS (27, 28), including increased ventricular wall tension from myocardial infarction, which stimulates NT-proBNP synthesis, and hypoxia caused by ACS, which can activate the cardiac endocrine system, resulting in increased NT-proBNP synthesis (29–31). NT-proBNP levels at baseline have been proved the strongest prognostic power independent of clinical predictors and demonstrated superior results to GRACE and thrombolysis in myocardial infarction (TIMI) risk scores in a validation cohort in a multicenter prospective study (32). Blood NT-proBNP levels at admission are not only practical, but they also have considerable predictive ability for the risk of long-term cardiovascular events.

Furthermore, ACS is significantly linked to a history of hypertension and high SBP (33–35). Evidence from both univariate and multivariate analyses points to a history of hypertension [OR = 2.62 (1.47–4.68), *p* = 0.001] and SBP [OR = 1.02 (1.01–1.04), *p* = 0.006] as risk factors for RCAL. According to Rao et al. (36), there is a linear association between systolic and diastolic blood pressure and cardiovascular outcomes, with each 10 mmHg rise in SBP increasing the risk of cardiovascular disease by 49%, coronary artery disease by 50%, and stroke by 44%. As a modifiable risk factor for ACS, SBP significantly affects the prognosis of patients with ACS (37). The SPRINT study (38) showed that compared to lowering SBP to less than 140 mmHg, controlling SBP to less than 120 mmHg significantly reduces the risk of MACEs, cardiovascular mortality, and all-cause mortality in patients at high cardiovascular risk.

Additionally, our study showed that the number of coronary vascular lesions and QRS intervals were independent risk factors for RCAL. SHAP analysis confirmed their impact on the prediction model. Typically, a higher prevalence of coronary vascular lesions often indicates that more heart areas are at risk of ischemia, which increases the risk of cardiac events and accelerates heart function decline. Pasceri et al. (39) discovered that comprehensive revascularization at the first PCI could reduce the total mortality and incidence of myocardial infarction compared to criminal coronary revascularization alone. However, staged revascularization did not substantially enhance outcomes. Thus, while assessing coronary progress in ACS patients following PCI, it is crucial to take the number of vascular lesions into special consideration.

Previous research has shown that QRS interval is indicative of left ventricular systolic dysfunction and poor outcomes such as heart failure OR death in myocardial infarction patients (40, 41). Our findings further identified QRS as an independent risk factor in the development of RCAL [OR = 1.02 (1.01–1.04), *p* = 0.001]. What's more, our study confirmed that age, history of hyperlipidemia, LAD, LVEF, IVDd, and RVDd were important factors in the RCAL prediction model. These factors have been shown to be closely related to coronary lesions and MACEs of ACS (42–47).

The RF model was employed to predict RCAL using the specified feature variables, demonstrating steady performance in external test cohorts, and shown robust generalization capability in predicting RCAL. Based on these findings, we created OpRCAL, a public online platform that facilitates personalized prediction of RCAL in patients with ACS after PCI.

We also acknowledge several limitations. First, this retrospective cohort study enrolled patients from two separate centers: one served as the training cohort and the other as an external test cohort. To better demonstrate the stability and generalizability of the prediction model, additional centers and a broader spectrum of clinical characteristics should be included, such as more details on PCI. Second, owing to the inherent quality issues of retrospective data, some key clinical features (e.g., troponin) were excluded from model development because of heterogeneous assay methods. Finally, although RF performed best among the nine ML algorithms examined, we did not investigate further algorithms or their combinations, which could be addressed in future prospective studies.

## 5 Conclusions

In patients with ACS, Lasso regression efficiently and effectively screened clinical variables linked to coronary progression following PCI. The RF model displayed commendable performance and robust generalization capabilities in predicting RCAL, potentially serving as a practical and important instrument for clinical decision-making in the domain of ACS.

## Data Availability

The raw data supporting the conclusions of this article will be made available by the authors, without undue reservation.

## References

[1] Nagasawa A Otake H Kawamori H Toba T Sugizaki Y Takeshige R Relationship among clinical characteristics, morphological culprit plaque features, and long-term prognosis in patients with acute coronary syndrome. Int J Cardiovasc Imaging. (2021) 37:2827–37. 10.1007/s10554-021-02252-w 33982195

[2] Fox KAA Anderson FA Goodman SG Steg PG Pieper K Quill A Time course of events in acute coronary syndromes: implications for clinical practice from the GRACE registry. Nat Clin Pract Cardiovasc Med. (2008) 5:580–9. 10.1038/ncpcardio1302 18665136

[3] Fox KAA Carruthers KF Dunbar DR Graham C Manning JR De Raedt H Underestimated and under-recognized: the late consequences of acute coronary syndrome (GRACE UK-Belgian study). Eur Heart J. (2010) 31:2755–64. 10.1093/eurheartj/ehq326 20805110

[4] Ridker PM. Residual inflammatory risk: addressing the obverse side of the atherosclerosis prevention coin. Eur Heart J. (2016) 37:1720–2. 10.1093/eurheartj/ehw024 26908943

[5] Cancro FP Bellino M Silverio A Di Maio M Esposito L Palumbo R Novel targets and strategies addressing residual cardiovascular risk in post-acute coronary syndromes patients. Transl Med UniSa. (2024) 26:99–110. 10.37825/2239-9747.1058 39385797 PMC11460530

[6] Giustino G Colombo A Camaj A Yasumura K Mehran R Stone GW Coronary in-stent restenosis: JACC state-of-the-art review. J Am Coll Cardiol. (2022) 80:348–72. 10.1016/j.jacc.2022.05.017 35863852

[7] Stone GW Maehara A Lansky AJ de Bruyne B Cristea E Mintz GS A prospective natural-history study of coronary atherosclerosis. N Engl J Med. (2011) 364:226–35. 10.1056/NEJMoa1002358 21247313

[8] Rossini R Oltrona Visconti L Musumeci G Filippi A Pedretti R Lettieri C A multidisciplinary consensus document on follow-up strategies for patients treated with percutaneous coronary intervention. Catheter Cardiovasc Interv. (2015) 85:E129–139. 10.1002/ccd.25724 25380511

[9] Wang H Gao Z Song Y Tang X Xu J Jiang P Impact of diabetes mellitus on percutaneous coronary intervention in Chinese patients: a large single-center data. Angiology. (2018) 69:540–7. 10.1177/0003319717735226 29073786

[10] Li J Han Y Jing J Tu S Chen W Reiber JHC Non-culprit coronary lesions in young patients have higher rates of atherosclerotic progression. Int J Cardiovasc Imaging. (2015) 31:889–97. 10.1007/s10554-015-0635-9 25749848

[11] Sakamoto I Mohri M Yamamoto H. Images in cardiovascular medicine. Rapid progression of coronary atherosclerosis by coronary artery spasm leading to acute coronary syndrome. Circulation. (2009) 119:2233–4. 10.1161/CIRCULATIONAHA.108.839522 19398679

[12] Kleppe A Skrede O-J De Raedt S Liestøl K Kerr DJ Danielsen HE. Designing deep learning studies in cancer diagnostics. Nat Rev Cancer. (2021) 21:199–211. 10.1038/s41568-020-00327-9 33514930

[13] Cilloniz C Ward L Mogensen ML Pericàs JM Méndez R Gabarrús A Machine-learning model for mortality prediction in patients with community-acquired pneumonia: development and validation study. Chest. (2023) 163:77–88. 10.1016/j.chest.2022.07.005 35850287

[14] Pacal I Attallah O. InceptionNeXt-transformer: a novel multi-scale deep feature learning architecture for multimodal breast cancer diagnosis. Biomed Signal Process Control. (2025) 110:108116. 10.1016/j.bspc.2025.108116

[15] Aruk I Pacal I Toprak AN. A novel hybrid ConvNeXt-based approach for enhanced skin lesion classification. Expert Syst Appl. (2025) 283:127721. 10.1016/j.eswa.2025.127721

[16] Attallah O Ma X Sedky M. Editorial: the role of artificial intelligence technologies in revolutionizing and aiding cardiovascular medicine. Front Cardiovasc Med. (2025) 12:1588983. 10.3389/fcvm.2025.1588983 40255343 PMC12006174

[17] Kaski JC Chester MR Chen L Katritsis D. Rapid angiographic progression of coronary artery disease in patients with angina pectoris. The role of complex stenosis morphology. Circulation. (1995) 92:2058–65. 10.1161/01.cir.92.8.2058 7554182

[18] Zouridakis EG Schwartzman R Garcia-Moll X Cox ID Fredericks S Holt DW Increased plasma endothelin levels in angina patients with rapid coronary artery disease progression. Eur Heart J. (2001) 22:1578–84. 10.1053/euhj.2000.2588 11492987

[19] Wang J Xu Y Liu L Wu W Shen C Huang H Comparison of LASSO and random forest models for predicting the risk of premature coronary artery disease. BMC Med Inform Decis Mak. (2023) 23:297. 10.1186/s12911-023-02407-w 38124036 PMC10734117

[20] Hadanny A Shouval R Wu J Gale CP Unger R Zahger D Machine learning-based prediction of 1-year mortality for acute coronary syndrome✰. J Cardiol. (2022) 79:342–51. 10.1016/j.jjcc.2021.11.006 34857429

[21] Davì G Patrono C. Platelet activation and atherothrombosis. N Engl J Med. (2007) 357:2482–94. 10.1056/NEJMra071014 18077812

[22] Mendelsohn ME Loscalzo J. Role of platelets in cholesteryl ester formation by U-937 cells. J Clin Invest. (1988) 81:62–8. 10.1172/JCI113311 3335643 PMC442473

[23] Chandler AB Hand RA. Phagocytized platelets: a source of lipids in human thrombi and atherosclerotic plaques. Science. (1961) 134:946–7. 10.1126/science.134.3483.946 13692295

[24] Barrett TJ Schlegel M Zhou F Gorenchtein M Bolstorff J Moore KJ Platelet regulation of myeloid suppressor of cytokine signaling 3 accelerates atherosclerosis. Sci Transl Med. (2019) 11:eaax0481. 10.1126/scitranslmed.aax0481 31694925 PMC6905432

[25] Natriuretic Peptides Studies Collaboration null, Willeit P Kaptoge S Welsh P Butterworth A Chowdhury R Natriuretic peptides and integrated risk assessment for cardiovascular disease: an individual-participant-data meta-analysis. Lancet Diabetes Endocrinol. (2016) 4:840–9. 10.1016/S2213-8587(16)30196-6 27599814 PMC5035346

[26] Lindahl B Lindbäck J Jernberg T Johnston N Stridsberg M Venge P Serial analyses of N-terminal pro-B-type natriuretic peptide in patients with non-ST-segment elevation acute coronary syndromes: a fragmin and fast revascularisation during in stability in coronary artery disease (FRISC)-II substudy. J Am Coll Cardiol. (2005) 45:533–41. 10.1016/j.jacc.2004.10.057 15708700

[27] Morrow DA de Lemos JA Blazing MA Sabatine MS Murphy SA Jarolim P Prognostic value of serial B-type natriuretic peptide testing during follow-up of patients with unstable coronary artery disease. JAMA. (2005) 294:2866–71. 10.1001/jama.294.22.2866 16352794

[28] Lindholm D James SK Gabrysch K Storey RF Himmelmann A Cannon CP Association of multiple biomarkers with risk of all-cause and cause-specific mortality after acute coronary syndromes: a secondary analysis of the PLATO biomarker study. JAMA Cardiol. (2018) 3:1160–6. 10.1001/jamacardio.2018.3811 30427997 PMC6583102

[29] Omland T Persson A Ng L O’Brien R Karlsson T Herlitz J N-terminal pro-B-type natriuretic peptide and long-term mortality in acute coronary syndromes. Circulation. (2002) 106:2913–8. 10.1161/01.cir.0000041661.63285.ae 12460871

[30] Wang J Gao W Chen G Chen M Wan Z Zheng W Biomarker-based risk model to predict cardiovascular events in patients with acute coronary syndromes - results from BIPass registry. Lancet Reg Health West Pac. (2022) 25:100479. 10.1016/j.lanwpc.2022.100479 35664511 PMC9160492

[31] Daniels LB Clopton P deFilippi CR Sanchez OA Bahrami H Lima JAC Serial measurement of N-terminal pro-B-type natriuretic peptide and cardiac troponin T for cardiovascular disease risk assessment in the multi-ethnic study of atherosclerosis (MESA). Am Heart J. (2015) 170:1170–83. 10.1016/j.ahj.2015.09.010 26678639 PMC4684596

[32] Jia X Al Rifai M Hoogeveen R Echouffo-Tcheugui JB Shah AM Ndumele CE Association of long-term change in N-terminal pro-B-type natriuretic peptide with incident heart failure and death. JAMA Cardiol. (2023) 8:222–30. 10.1001/jamacardio.2022.5309 36753229 PMC9909572

[33] Pedrinelli R Ballo P Fiorentini C Denti S Galderisi M Ganau A Hypertension and acute myocardial infarction: an overview. J Cardiovasc Med (Hagerstown). (2012) 13:194–202. 10.2459/JCM.0b013e3283511ee2 22317927

[34] Perry HM Davis BR Price TR Applegate WB Fields WS Guralnik JM Effect of treating isolated systolic hypertension on the risk of developing various types and subtypes of stroke: the systolic hypertension in the elderly program (SHEP). JAMA. (2000) 284:465–71. 10.1001/jama.284.4.465 10904510

[35] Amar J Chamontin B Ferriéres J Danchin N Grenier O Cantet C Hypertension control at hospital discharge after acute coronary event: influence on cardiovascular prognosis–the PREVENIR study. Heart. (2002) 88:587–91. 10.1136/heart.88.6.587 12433885 PMC1767443

[36] Rao S Li Y Nazarzadeh M Canoy D Mamouei M Hassaine A Systolic blood pressure and cardiovascular risk in patients with diabetes: a prospective cohort study. Hypertension. (2023) 80:598–607. 10.1161/HYPERTENSIONAHA.122.20489 36583386 PMC9944753

[37] Deng Y Liu Y Yang L Bai J Cai J. Improving outcomes for older hypertensive patients: is more intensive treatment better? Expert Rev Cardiovasc Ther. (2022) 20:193–205. 10.1080/14779072.2022.2058491 35332819

[38] SPRINT Research Group, Wright JT Williamson JD Whelton PK Snyder JK Sink KM A randomized trial of intensive versus standard blood-pressure control. N Engl J Med. (2015) 373:2103–16. 10.1056/NEJMoa1511939 26551272 PMC4689591

[39] Pasceri V Patti G Pelliccia F Gaudio C Speciale G Mehran R Complete revascularization during primary percutaneous coronary intervention reduces death and myocardial infarction in patients with multivessel disease: meta-analysis and meta-regression of randomized trials. JACC Cardiovasc Interv. (2018) 11:833–43. 10.1016/j.jcin.2018.02.028 29747913

[40] Kurl S Mäkikallio TH Rautaharju P Kiviniemi V Laukkanen JA. Duration of QRS complex in resting electrocardiogram is a predictor of sudden cardiac death in men. Circulation. (2012) 125:2588–94. 10.1161/CIRCULATIONAHA.111.025577 22615341

[41] López-Castillo M Aceña Á Pello-Lázaro AM Viegas V Merchán Muñoz B Carda R Prognostic value of initial QRS analysis in anterior STEMI: correlation with left ventricular systolic dysfunction, serum biomarkers, and cardiac outcomes. Ann Noninvasive Electrocardiol. (2021) 26:e12791. 10.1111/anec.12791 32845542 PMC7816810

[42] Mohammad MA Stone GW Koul S Olivecrona GK Bergman S Persson J On the natural history of coronary artery disease: a longitudinal nationwide serial angiography study. J Am Heart Assoc. (2022) 11:e026396. 10.1161/JAHA.122.026396 36300820 PMC9673651

[43] Gallone G Kang J Bruno F Han J-K De Filippo O Yang H-M Impact of left ventricular ejection fraction on procedural and long-term outcomes of bifurcation percutaneous coronary intervention. Am J Cardiol. (2022) 172:18–25. 10.1016/j.amjcard.2022.02.015 35365291

[44] Ri T Saito C Arashi H Yamaguchi J Ogawa H Hagiwara N. Increased left atrial volume index is associated with more cardiovascular events in patients with acute coronary syndrome: HIJ-PROPER study findings. Echocardiography. (2022) 39:260–7. 10.1111/echo.15301 35043458

[45] De Filippo O D’Ascenzo F Wańha W Leonardi S Raposeiras Roubin S Fabris E Incidence and predictOrs of heaRt fAiLure after acute coronarY syndrome: the CORALYS registry. Int J Cardiol. (2023) 370:35–42. 10.1016/j.ijcard.2022.10.146 36306949

[46] Huang B-T Peng Y Liu W Zhang C Huang F-Y Wang P-J Increased interventricular septum wall thickness predicts all-cause death in patients with coronary artery disease. Intern Med J. (2015) 45:275–83. 10.1111/imj.12667 25510963

[47] Hamon M Agostini D Le Page O Riddell JW Hamon M. Prognostic impact of right ventricular involvement in patients with acute myocardial infarction: meta-analysis. Crit Care Med. (2008) 36:2023–33. 10.1097/CCM.0b013e31817d213d 18552681

