# Supplementary material for: Predicting the rapid progression of coronary artery lesions in patients with acute coronary syndrome based on machine learning
Source: Front Cardiovasc Med. 2025 Sep 5;12:1535406. doi: 10.3389/fcvm.2025.1535406 (PMC12447524; doi:10.3389/fcvm.2025.1535406)
Supplement: Supplementary file 1 [file Datasheet1.docx]

**Supplementary material**

**Table S1 Univariate and multifactorial analysis of rapid progression of coronary artery disease.**

| **Variable** | **Univariate analysis** | |  | **Multivariate analysis** | |
| --- | --- | --- | --- | --- | --- |
|  | **OR (95%CI)** | ***P*-value** |  | **OR (95%CI)** | ***P*-value** |
| History of hypertension |  |  |  |  |  |
| No | 1.00 (Reference) | - |  | - | - |
| Yes | 2.62 (1.47 -4.68) | 0.001 |  | - | - |
| Receiving ACEI |  |  |  |  |  |
| No | 1.00 (Reference) | - |  | - | - |
| Yes | 2.21 (1.16 -4.22) | 0.016 |  | - | - |
| Number of coronary lesions | 1.64 (1.22 -2.20) | <0.001 |  | 1.55 (1.09 -2.20) | 0.014 |
| SBP | 1.02 (1.01 -1.04) | <0.001 |  | 1.02 (1.01 -1.04) | 0.006 |
| NT-proBNP | 1.01 (1.01 -1.01) | <0.001 |  | 1.01 (1.01 -1.01) | 0.001 |
| LVEF | 0.95 (0.92 -0.98) | <0.001 |  | - | - |
| LAD | 1.12 (1.05 -1.19) | <0.001 |  | - | - |
| LVDd | 1.06 (1.01 -1.12) | 0.042 |  | - | - |
| LVPWd | 1.31 (1.08 -1.58) | 0.005 |  | - | - |
| IVSd | 1.25 (1.08 -1.45) | 0.003 |  | - | - |
| RVDd | 1.17 (1.04 -1.31) | 0.010 |  | - | - |
| QRS interval | 1.02 (1.01 -1.03) | 0.025 |  | 1.02 (1.01 -1.04) | 0.034 |
| PLT | 1.02 (1.02 -1.03) | <0.001 |  | 1.02 (1.02 -1.03) | <0.001 |
| hs-CRP | 1.01 (1.01 -1.02) | 0.033 |  | - | - |

Abbreviations: *OR,* odds ratio; *CI,* confidence interval; *ACEI,* angiotensin converting enzyme inhibitors; *SBP,* systolic blood pressure; *NT-proBNP,* N-terminal pro-brain natriuretic peptide; *LVEF,* left ventricular ejection fraction; *LAD*, left atrium diameter; *LVDd,* left ventricular end-diastolic diameter; *LVPWd,* left ventricular posterior wall diastolic thickness; *IVSd,* interventricular septal end-diastolic thinkness; *RVDd,* right ventricular end-diastolic diameter; *PLT,* platelet; *hs-CRP,* high sensitivity C reactive protein.

.

| **Variable** | **Tolerance** | **VIF** |
| --- | --- | --- |
| Age | 0.903 | 1.108 |
| History of hypertension | 0.767 | 1.303 |
| History of hyperlipidemia | 0.961 | 1.040 |
| SBP | 0.808 | 1.238 |
| Number of coronary lesions | 0.955 | 1.048 |
| NT-proBNP | 0.872 | 1.147 |
| LVEF | 0.777 | 1.286 |
| LAD | 0.692 | 1.444 |
| IVSd | 0.798 | 1.253 |
| RVDd | 0.773 | 1.294 |
| QRS interval | 0.917 | 1.090 |
| PLT | 0.946 | 1.057 |

**Table S2 Variance inflation factors (VIFs) between variables.**

Abbreviations: *SBP,* systolic blood pressure; *NT-proBNP,* N-terminal pro-brain natriuretic peptide; *LVEF,* left ventricular ejection fraction; *LAD,* left atrium diameter; *IVSd,* interventricular septal end-diastolic thinkness; *RVDd,* right ventricular end-diastolic diameter; *PLT,* platelet.


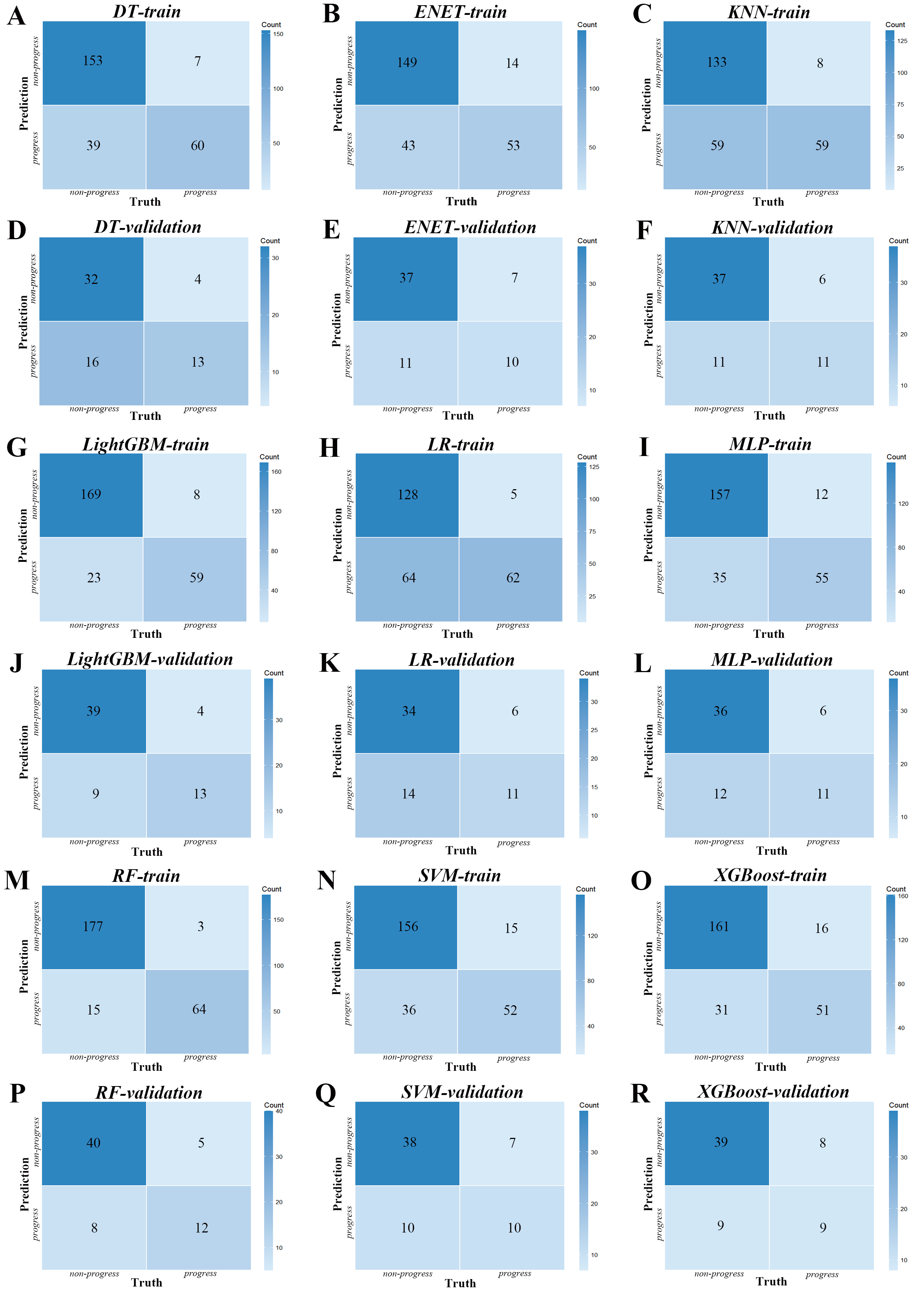


**Fig. S** Confusion matrix in constructing predictive models using 9 machine learning algorithms. **A-C, G-I, M-O**: Confusion matrix of DT, ENET, KNN, LightGBM, LR, MLP, RF, SVM, and XGBoost in the internal training cohort, **D-F, J-L, P-I**: Confusion matrix of DT, ENET, KNN, LightGBM, LR, MLP, RF, SVM, and XGBoost in the internal validation cohort.

Abbreviations: *DT,* decision trees; *ENET,* elastic net; *KNN,* k-nearest neighbor; *LightGBM,* light gradient boosting machine; *LR,* logistic regression; *MLP,* multilayer perceptron; *RF,* random forest; *SVM,* support vector machine; *XGBoost,* extreme gradient boosting.
